# Knowledge is power: general practitioners prescribing of new oral anticoagulants in Ireland

**DOI:** 10.1186/s13104-018-3597-x

**Published:** 2018-07-16

**Authors:** A. Murphy, A. Kirby, C. Bradley

**Affiliations:** 1 Department of Economics, Cork University Business School, University College Cork, Cork, Ireland; 2 Department of General Practice, College of Medicine, University College Cork, Cork, Ireland

**Keywords:** Anticoagulation, Prescribing decisions, Education, Primary care

## Abstract

**Objective:**

New oral anticoagulants (NOACs) aim to overcome warfarin’s shortcomings, however their pharmacokinetic characteristics make prescribing complex. Thus it is imperative that general practitioners (GPs) are aware of specific treatments so as to maximise their benefits and minimise their pitfalls. This study explores GPs attitudes and experiences with prescribing NOACs in Ireland where, despite clear national prescribing guidelines advocating warfarin as first line therapy, the number of patients being prescribed NOACs for the first time is growing.

**Results:**

Using primary data collected from GPs in Ireland the factors influencing the likelihood of a GP initiating a prescription for a NOAC are determined using a probit model. Results indicate 46% of the sample initiated NOAC prescriptions and GP practice size is a significant factor influencing this. Analysis revealed no difference regarding the sources of information considered important amongst GPs when prescribing new drugs. However, there were differences in which factors were considered important when prescribing anticoagulants between initiating and non-initiating NOAC prescribers. The results of this study suggest better utilisation of existing information and education tools for GPs prescribing NOACs and managing NOAC patients is imperative, to ensure the right anticoagulant is prescribed for the right patient at the right time.

## Introduction

Atrial fibrillation (AF) is an abnormal heart rhythm. Owing to risk of thromboembolism, patients with AF have a five-fold increase in risk of having a stroke [1] and higher mortality and recurrence [2]. To prevent stroke and thromboembolism AF patients are typically administered an anticoagulant. Warfarin, the original anticoagulant, has been prescribed for decades worldwide [3]. Recently four new anticoagulants acquired European Medicines Agency and Food and Drug Administration approval: dabigatran, rivaroxaban, apixaban and edoxaban. They are collectively referred to as new oral anticoagulants (NOACs), and more recently direct oral anticoagulants (DOACs).

Despite its dominance, those on warfarin are at risk of bleeding, consequently extensive monitoring is required owing to its narrow therapeutic range, which is expensive and time consuming [4]. NOACs have broadly similar efficacy and safety compared with warfarin and are thus emerging as alternatives for the prevention of stroke and embolic events in AF patients [5]. Nevertheless, there are pharmacology differences amongst NOACs, as well as differences in the trial designs and outcomes on which their potential is based. These result in challenging clinical issues [5–7] which need to be understood and considered by prescribers. Furthermore, NOACs are expensive so to achieve value for money it is imperative that they are prescribed appropriately.

NOACs have been adopted rapidly in the US and other jurisdictions, but some health authorities have been more cautious. For example, in Ireland even though each NOAC is considered cost effective, warfarin remains the preferred anticoagulant for stroke prevention amongst non-valvular AF patients, followed by apixaban [8]. Yet NOACs are increasingly prescribed for AF patients. Between January 2013 and March 2014, 8399 patients were prescribed NOACs for the first time [8] and in 2014 expenditure on NOACs represented 88% of total annual spend on anticoagulants [9].

The range of influences on NOAC prescribing is diverse. Huang et al. [10] found dabigatran prescribing is influenced by cost, safety and effectiveness of dabigatran; patients’ previous exposure; adherence and experience with warfarin; as well as patient preferences [10]. Further studies are needed to investigate NOAC prescribing patterns [11].

This study examines the factors that influence general practitioners (GPs) when initiating NOAC prescribing in Ireland. While a multi-disciplinary approach is advocated to prescribing NOACs, the GP is often patients’ first point of contact. Consequently, local leadership and expert champions amongst GPs are advocated [12, 13].

Ireland was chosen as a case study for this analysis owing to the relatively conservative approach to prescribing NOACs being advocated by authorities. Furthermore, as Ireland features a mix of public and private patients it has features of both the social insurance and private health care systems. In Ireland the state pays for approximately 80% of all medicines through general taxation. The remaining 20% is funded through out of pocket payments. Through means-tested schemes GP visits and drugs are paid for or subsidized in the community. In addition, for patients ineligible for existing schemes there is a separate NOAC reimbursement scheme [14]. Thus there is little incentive for public patients to take into account price differences and no income incentive on the physicians’ choice of drug.

## Main text

### Methods

A dedicated postal survey was designed, piloted and disseminated to Irish GPs in November 2015. Ethical approval was acquired from the University’s Social Research Ethics Committee. There are approximately 2500 practicing GPs in Ireland (combination of private and public practice) [15]. As a current comprehensive list of GPs was not available as a sampling frame, the telephone directory was used to compile a listing of over 1422 GPs (as per Bourke and Bradley [16]). Excluding incomplete surveys the sample size was 221 (15%).

A probit model was employed to determine the factors influencing the likelihood of a GP initiating a prescription for a NOAC (StataCorp LP). This accommodated a binary dependent variable with a separation between GPs who initiated prescribing of new oral anticoagulants and GPs who did not. Here the dependent Y represents an initiating prescriber of NOACs/non-initiating prescriber of NOACs. Included is a vector of regressors $\varvec{X}^{T}$ which influences the outcome variable (includes gender, age, number employed in practice, location of the practice, services provided at the practice (anticoagulation, diet, counsellor and physiotherapy clinics), dispensing service and if the practice is a GP training practice).

$$\Pr \left( {Y = 1|X} \right) = \phi (\varvec{X}^{T} \beta )$$

where Pr denotes the probability, $\phi$ is the cumulative distribution function of the standard normal distribution. The parameters that are included in $\beta$ are estimated by maximum likelihood. Marginal Effects were also estimated and presented.

### Results

#### Important factors influencing initial prescribers of NOACs

All respondents had patients currently prescribed an anticoagulant for AF. 70% of GPs were male, aged 53 years on average. Over half the respondents are based in GP practices in the southern region of Ireland (Munster) and 61% in a city or large town. With regard to practice size, the average number of public patients per practice is 1681 and private patients is 2037. Most practices employ a mix of full and part time staff, with five staff members on average. 36% reported having anticoagulation clinics and 10% reported having other clinics. Table 1 presents the summary statistics.

**Table 1:** Summary statistics: GP practices in Ireland

Variable name	Full sample (N = 221)	Initiating prescribers (N = 101)	Non initiating prescribers (N = 120)
	Mean (SD)	Mean (SD)	Mean (SD)
Age	53 (10.23)	52 (9.71)	53 (10.66)
No. of GMS	1681 (1380)	1972 (1439)	1427 (1279)
No. of private	2037 (2205)	2472 (2583)	1658 (1738)
Size of the practice	3616 (3148)	4345 (3662)	2988 (2476)
No. of employees	5 (4)	5 (4)	5 (4)

	Freq (%)	Freq (%)	Freq (%)
Males	154 (70)	74 (73)	80 (67)
Munster	108 (49)	53 (52)	55 (46)
Leinster	63 (29)	25 (25)	38 (32)
Connaght	33 (15)	17 (17)	16 (13)
Ulster	10 (5)	6 (6)	8 (7)
City	69 (31)	24 (24)	45 (37)
Large Town (3000–10,000)	67 (30)	34 (34)	33 (27)
Small town (800–3000)	45 (20)	29 (29)	16 (13)
Village (< 800)	28 (14)	10 (10)	18 (15)
Rural	12 (5)	4 (3)	8 (7)
Anticoagulation clinic	7 (3)	4 (4)	3 (2)
Dispensing clinic	15 (7)	8 (8)	7(6)
Physiotherapy clinic	44 (2)	17 (17)	27 (22)
Diet clinic	40 (18)	17 (17)	23 (17)
GP training practice	73 (33)	37 (37)	36 (30)

Provinces aggregate to 214 due to non-response. Size of practice, No. of GMS and Private patients have 5, 6 and 15 missing values respectively

The survey differentiated between GPs who had, themselves, initiated NOAC prescribing (46%) and those who had not (54%). (The latter do prescribe NOACs but only when prescribing has been initiated by another clinician).

Amongst the full sample, hospital consultants (92%), other GPs (86%), conferences (83%) and journals (79%) were ranked as important influences for GPs when prescribing new drugs. Pharmaceutical representatives (67%) and pharmaceutical representatives at events (54%) and the internet (50%) were ranked as not important (aggregate of responses 1 or 2). The order of these rankings was the same amongst initiating prescribers.

**Fig. 1:**
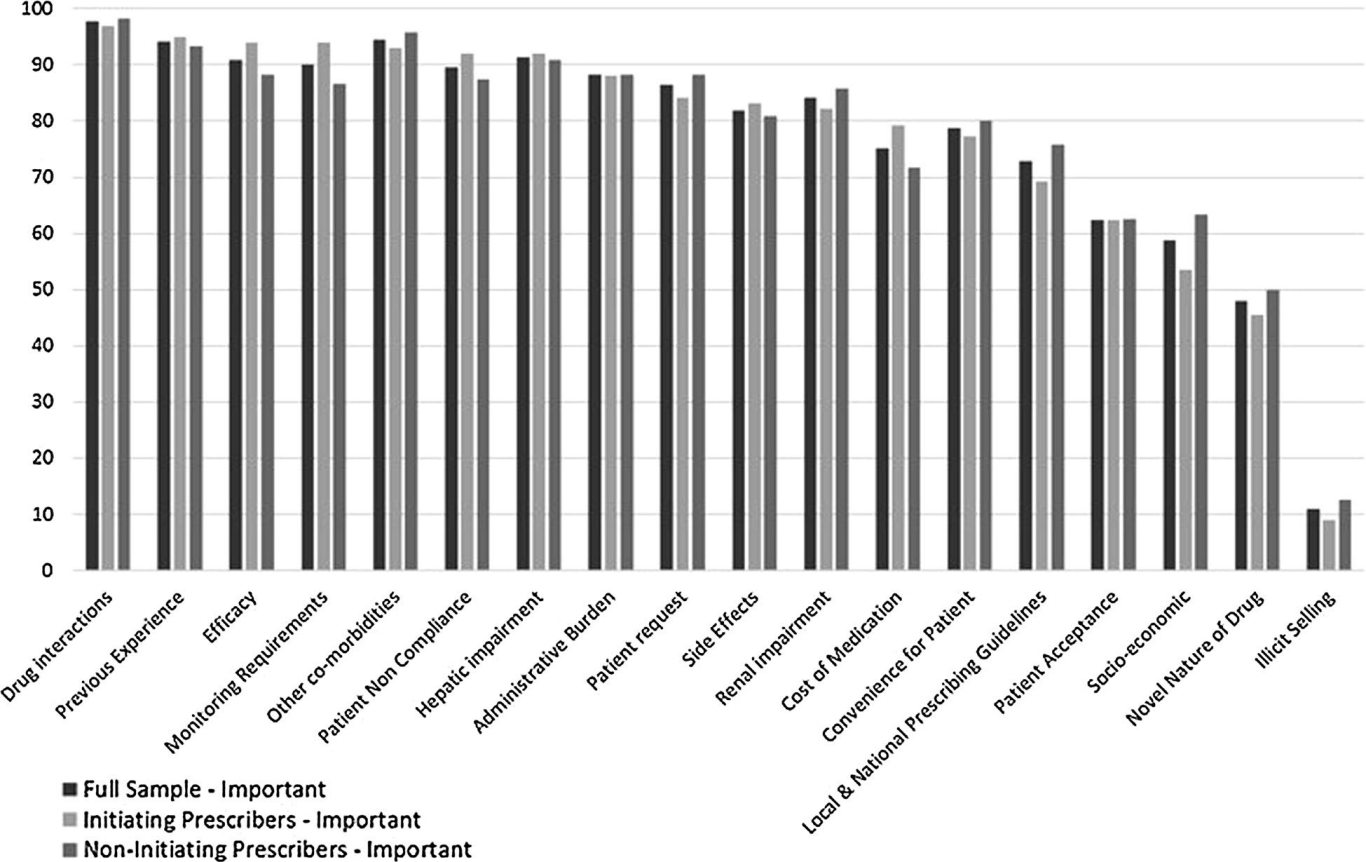
Important factors influencing NOAC prescribing. Note respondents were asked to rank the importance of each factors from 1 to 5, where 1 was not important and 5 was very important. Important here is the aggregate of responses equal to 3, 4 and 5

Respondents were also asked to rank by importance the factors that influence their NOAC prescribing decision for patients with AF (Fig. 1). Over 90% of all respondents indicated that drug interactions, previous experience with the drug, efficacy, monitoring requirements, patient non-compliance, other co-morbidities and hepatic impairment were important factors to consider when prescribing NOACs. Renal impairment was only ranked as important by 84% of the sample, cost of the medication by 75% of the total sample and socio-economic factors were ranked as important by 59% of the sample.

Comparing factor rankings between initiating prescribers of NOACs (n = 101) and non-initiating prescribers (n = 120) there are few differences. Over 90% of initiating prescribers ranked drug interactions, previous experience, efficacy, monitoring requirements, other co-morbidities, patient non-compliance, and hepatic impairment as important. Over 80% ranked, administrative burden, patient request, side effects and renal impairment as important. However, only 82% of initiating prescribers ranked renal impairment as important compared with 84% of the full sample. In the full sample, prescribers in 15 practices ranked all three factors (i.e., renal impairment, hepatic impairment and other co-morbidities) as not important (i.e., ranked all three factors 1 or 2). As these account for approximately 95 patients on NOACs this is cause for concern. Meanwhile amongst non-initiating prescribers only 87% ranked monitoring requirements as important, while only 88% ranked patient non-compliance as important. Interestingly, more non-initiating prescribers (76% versus 69%) ranked local and national guidelines as important.

#### GP practice characteristics influencing prescribers who initiate NOAC prescriptions

The probit model was used to determine the factors influencing the probability of a GP initiating a NOAC prescription. The dependent variable was initiating or non-initiating prescribers of NOACs. Practice size was measured by the number of employees in the model and the location of the practice was measured using a dummy variable for urban and rural areas. Correlation between age and years practicing as a GP was expected and confirmed (0.88) so to avoid multicollinearity only age was included. The marginal effect was estimated at the mean. The probit results revealed that as practice size increased GPs were more likely to initiate NOAC prescriptions holding all other variables at the mean (Table 2). No other variables were found to be statistically significant.

**Table 2:** Probit results for initial prescribers

Variables	dy/dx	SE
Male	0.096	0.074
Age	− 0.001	0.003
No. of employees	0.024*	0.012
Urban	0.100	0.092
Anticoagulation clinic	0.097	0.256
Dispensing clinic	0.079	0.180
GP training practice	0.048	0.075
Physiotherapy clinic	− 0.098	0.092
Diet clinic	− 0.086	0.096
Counsellor clinic	0.026	0.084

*5% level of significance

### Discussion

While some of the concerns around NOACs are being resolved (increased monitoring and emergence of antidotes etc.) others persist owing to their pharmacokinetic and pharmacodynamic characteristics. As NOAC prescribing continues to grow their successful use relies on prescribers and those managing NOAC patients’ care understanding these considerations. This requires education and guidance to ensure; proper clinical management for monitoring [6, 17]; that the advantages and limitations of NOACs for prescribers are recognised [18]; that the management of complications and adverse events are known and appreciated [6, 19] and that physician knowledge and confidence is increased, specifically on the pharmacokinetic and pharmacodynamic characteristics of the agents [19].

The success of NOAC prescribing also requires integration between services, particularly between hospital consultants and GPs who are caring for NOAC patients. The survey results reported indicate that GPs are cognisant of this (hospital consultants are acknowledged an important source of information). Furthermore, the probit model results indicate that practice size is a positive influence on being an initial prescriber. This result could suggest that as a practice grows in size that there is more opportunity to share information from different sources.

With regard to important factors to consider when prescribing anticoagulants some knowledge gaps exist between prescribers and non-prescribers. For example, a greater share of those who are prescribing NOACs acknowledged patient non-compliance and the need for some type of monitoring. Given the differences in half-life between different NOACs these are important considerations when treating patients prescribed NOACs or when prescribing NOACs for the first time. Furthermore, about 16% of the full sample and 18% of GPs initiating prescribing considered renal impairment as not-important; while 6% of GPs in the full sample and 4% of initiating prescribers considered hepatic impairment as not important when prescribing NOACs. Given the emphasis placed on these two factors by the European Heart Rhythm Association [20] and Irish College of General Practitioners [21] guidelines this is worrying. It suggests better dissemination of and greater emphasis on existing information tools, such as the “Anticoagulation Prescribing Tips” guide [8], to inform prescribers as suggested in the literature [6, 18, 19]. Moreover, this suggests that experience with prescribing warfarin is not a substitution for knowledge and education about NOACs. Also, the differences between initiating and non-initiating prescribers on the importance of factors like monitoring, patient compliance etc., while subtle, suggests response bias may be concealing the true depth of knowledge gaps.

### Conclusions

The growth of NOACs is unsurprising and their availability generates choice for patients and prescribers. Nevertheless concerns persist: delays in the development of antidotes or reversal agents; medication adherence; higher cost of the NOACs compared to warfarin; lack of standardised and available tests to monitor/assess the level of anticoagulant activity; contraindications and drug interactions [22].

As a result, making the best NOAC prescribing decisions, in such a complex environment with many influences, is challenging. While the factors influencing decisions may change over time as new and pragmatic evidence emerges, it is imperative that GPs prescribing NOACs are aware of and utilise the existing prescribing guidance so as to maximise the benefits and avoid the pitfalls of NOACs [23].

## Limitations

While postal surveys are frequently used to collect information from physicians, owing to their ease of use and relatively inexpensive nature we acknowledge that they are subject to limitations such as a low response rate and response bias [24–26]. Nevertheless the representativeness of the sample does appear to be in line with previous studies [16, 27] and OECD statistics [28].

## Abbreviations

AF: atrial fibrillation
DOACs: direct oral anticoagulants
GPs: general practitioners
NOACs: new oral anticoagulants
